# The Correlation of Psychology and Physiology

**Published:** 1854-10-01

**Authors:** 


### Art. III.—THE CORRELATION OF PSYCHOLOGY AND PHYSIOLOGY.

Dr. Noble treats very concisely of weighty matters in the three Lectures which he has reprinted from the *Association Medical Journal.** The first is a summary of the Anatomy and Physiology of the Nervous System; the second considers "Emotional Sensibility and its Reactions;" the third, of "Ideas and their Dynamic Influence."

From the remotest periods of physiological speculation, the brain and nervous system, as Dr. Noble remarks, have been supposed to have some special connexion with the manifestations of conscious life. Hippocrates, in his treatise, "*De Morbo Sacro*," refers insanity as well as epilepsy to the brain; and to that extent his pathology is sound. He remarks, after referring epilepsy to the brain, "And by the same organ we become mad and delirious, and fears and terrors assail us, some by night and some by day, and dreams and untimely wanderings, and cares that are not suitable, and ignorance of present circumstances, desuetude and unskilfulness. All these things we endure from the brain, when it is not healthy, * * * and we become mad from humidity."* There are passages in the Roman writers which prove that then, as now, the brain was proverbially considered to be the seat of insanity. A quotation from Horace will show this:—

> "Si malè rem gerere insani est; contra, benè sani:
> Putidius multò cerebrum est (mihi crede) Perillî
> Dictantis, quod tu nunquam rescribere possis."

The Hippocratic theory of insanity is, in fact, condensed in the single word "putidius."

Modern research has given greater clearness to cerebral anatomy and physiology by researches conducted in the spirit of the inductive philosophy. It is now generally acknowledged that the nervous system is made up of two distinct portions—the one called the white matter, the elementary fibrils of which *conduct* the dynamical influence derived from the other, termed *grey*, or, more correctly, vesicular neurine. The latter is in the centres of the nervous system and in the peri-

* Three Lectures on the Correlation of Psychology and Physiology. By Daniel Noble, M.D., Visiting Physician to the Clifton Hall Retreat, near Manchester. Pamphlet, pp. 45. 1854.

† Dr. Adam's translation for the Sydenham Society, p. 855.

phery of the body, whether we include in that term the cutaneous, mucous, serous, or muscular tissues. Between these are the afferent and efferent nerves, or, as Unzer denominated them, the to-and-fro carrying nerves. A *suitable* impression acting upon the vesicular neurine distributed on the cutaneous or mucous surfaces, induces a continuous change upwards in the *ad*-ferent (the to-carrying) nerve fibrils; and this change acting in a pre-ordained way upon the central vesicular neurine—still as an impression—induces a change in the ef-ferent (the fro-carrying) nerve fibrils, which reaching to muscular or secernent tissues, excites motions or secretions adapted to the wants of the organism. The motions thus induced have been termed *reflex*, *excito-motory*, and *automatic*. "The purpose of the spinal axis and its reflex function would appear to be the conservation of the organism, through excitation of the respiratory acts, by its governance of the various orifices of ingress and egress, and by its contribution to the integrity of some other processes in which reflex movements participate." We quote Dr. Noble. With regard to the sympathetic system, he remarks, "It most likely communicates a susceptibility to certain motions involved in the processes of circulation, nutrition, and secretion; an influence not needed for their simple accomplishment, but required in the animal economy, in order that they may become related with, and in a measure subordinated to, the higher operations of the brain and nervous system." Dr. Noble has followed Dr. Hall in stating the functions of the spinal cord, or "true spinal system." We think, *mutatis mutandis*, that he would have found a better guide in Unzer—certainly one more systematic, clear, and philosophical. That author, we are satisfied, has not been *studied* (perhaps not read even) by modern neurologists, otherwise errors would have been avoided which Unzer has most clearly indicated. The nerves of *special* sense, as they are termed, that is to say, those through which man is enabled to take cognisance of the qualities and characteristics of external objects—smell, taste, hearing, sight, touch—are generally considered, as a matter of course, to be in constant relation to consciousness. Now, it is probable that without them consciousness cannot exist; but the converse is less probable, that they cannot be present and there not be consciousness. Unzer amply shows that external impressions will excite what appear to be volitional actions, but which are really "nerve actions"—the results of the operation of the *vis nervosa*, or, in modern phrase, reflex acts. They are excited (as he demonstrates by experiments), whether the animal feels them or not, and independently of any reference to the gratification of any desire or instinct. "The phenomena manifested by newly-born and decapitated animals, some of which have been already stated, amply prove that such apparently volitional acts may take place, under circumstances which altogether exclude the idea of mind. What in them appears to be volitional, only *appears* so, because we draw conclusions as to other animals from the nature and working of our own minds. What appears to be designed, arises from the pre-ordination of nature, and in no case enters into the minds of even sentient animals."*

These remarks are more especially applicable to the fifth sense, namely, "common sensation," in which Dr. Noble includes *touch*, and which has its proper nerves and its "grey vesicular centres—the ganglia of common sensation." Dr. Noble observes as to the latter—

"Physiologists are not agreed as to the identity of these structures; they must be expected, however, like the other sensory ganglia, to be somewhere at the base of the cranium; and I am myself disposed to think that the vesicular nuclei within the lateral lobes of the cerebellum constitute the encephalic centres of common sensation. Many years ago, Foville assigned this function to the aggregate cerebellum; and others with great plausibility have advocated the same notion. The anatomical connexion which exists between the ganglionic structures in question and the posterior columns of the spinal cord, through the corpora restiformia, favours the idea which I have advanced; and there are various physiological and pathological facts which go to corroborate it."

The experiments of Magendie and Lugol are quoted as showing that the slightest touch of the fibres of the restiform bodies induces violent pain. A case related by Hutin "in which the sense of touch was so exalted that, upon the least contact, intolerable pain and restlessness ensued, with corresponding muscular contractions, resembling those produced by an electric discharge;" and in which, after death, among other changes, atrophy of the cerebellum was found, is also noticed. Dr. Noble further thinks that this view is reconcilable in some degree with the phrenological doctrine as to the sexual relations of the cerebellum, and with that which appropriates to it the function of co-ordinating the various muscles into their infinitely varied combinations. Dr. Noble's hypothesis has the merit of ingenuity; we need hardly say that he does not esteem it, nor advance it, as more than an hypothesis.

All these sensory ganglia have a close relation with the muscular system, as well as with the consciousness. Dr. Noble remarks:—

"It would seem that impressions received in some particular ganglionic structure may be diffused through a whole chain of connected ganglia, and so bring about respondent movements of very varied character. These Dr. Carpenter designates *con-sensual*, not in the meaning of consentaneousness, but as occurring *with*, in dependence upon, *sense*. * * * An odious taste simply may determine the involuntary act

* Dr. Laycock's translation for the Sydenham Society, p. 323.

of vomiting; a loud and unexpected sound will occasion slight but very general contraction of the muscles, as in startling; the eye, when dazzled, is rapidly withdrawn from the light; and a sudden dash of cold water provokes deep inspiration and audible sobbing. These muscular actions are reflex as to their modes of occurrence; but they differ from the reflex actions purely spinal in being essentially attended with consciousness; and they differ from ordinary movements in the circumstance that neither volities, nor ideas, nor mental emotion, properly speaking, are concerned in their production."

We are of opinion that sensation or consciousness are not *necessarily* connected with these movements, so accurately and lucidly described by Dr. Noble, and therefore are not "dependent upon sense." Mere consciousness is a passive recipient state of the mind, and cannot excite muscular action; nor can that form of it termed sensation; it is the *will* which is the moving power mentally—the functional activity of the cerebro-spinal centres corporeally. It is already granted that the spinal cord on the one hand, and the hemispherical ganglia on the other, may be functionally active, independently of consciousness or sensation; and we cannot understand why the sensorial ganglia, as they are termed, should form an exception to that *grand common law of* ALL *organized matter*, whether it be animal or vegetable—namely, automatic unconscious, but admirably adapted, vital action on the reception of an appropriate stimulus. A study of the metaphysics (if we may be permitted the phrase) of *cell* life, whether animal or vegetable, will reveal *quasi* mental processes quite as definite as those carried on by the cells of the vesicular neurine, whether they constitute the spinal, sensorial, or hemispherical ganglia. The latter are nothing but organized matter, like the former, and have functions generically the same. The laws of functional activity of the one are applicable to the other. All may be active without consciousness or the co-operation of percipient mind. To this great and comprehensive conclusion (we are satisfied) neurologists and metaphysicians must ultimately come.

The second Lecture, "On Emotional Sensibility and its Reactions," is particularly interesting. Dr. Noble shall state his views in his own words:—

"There is a sensibility more elevated in the psychical scale than either external sensation or the physical appetites; I refer to that all-pervading sense of bodily existence which the German psychologists have named *Cœnæthesis*—general feeling, and sometimes self-feeling (Selbst-gefühl). This sensibility connects itself, apparently, with the peripheral termination of nerves throughout the whole body, but more particularly of those supplying the thoracic and abdominal viscera. It would seem to localize itself in an especial manner about the præcordial region. It will best be indicated psychologically by use of the popular phraseology, *the spirits*. Under ordinary circumstances, this senseconsciousness, is that of bodily contentment—tranquil spirits. When it is exalted, we are said to be in high spirits, glad at heart, joyous. * * * When it is depressed, low spirits are experienced; we are heavy and dull, and inapt for exertion. Acutely felt, it is *emotion*. These several states of the animal spirits, so designated, may result from purely physical causes, and in their origin be quite irrespective of thought."

Certain conditions of the viscera and atmospheric changes are well known for their influence on "the spirits." Morbid conditions of the blood, and of the nervous system itself, are also in the first class of physical agents. Dr. Noble very clearly distinguishes between this feeling of the state of the organism and common sensibility, in the perception of pain or pleasure through the *nerves*. It is an "emotive sense," with a suitable *habitat* in the encephalon. Dr. Noble differs from Dr. Carpenter as to the seat of this, as well as of common sensation. We have seen that he places the latter in the inferior ganglia of the cerebellum. Dr. Carpenter indicates the optic thalami connecting the corpora striata with it as the seat of the respondent movements. These, Dr. Noble thinks, "form the special region of emotional sensibility," adducing in proof arguments drawn from comparative anatomy, experimental vivisections, and pathological anatomy. "Certain nations," he adds, "are characterized more than others by emotional sensibility; the Irish, for example, more than the Scotch. Women are, in this respect, more remarkable than men. It would be interesting to compare the relative development of the optic thalami and corpora striata in the respective instances." Emotional sensibility, he thinks, induces its own reactions upon the muscular system, independently of the movements denominated con-sensual.

The third Lecture, "On Ideas and their Dynamic Influences," is the most important of the three. The automatic action of the cerebrum—its reflex function—is first illustrated.

"From the dominance of particular ideas, movements very often become excited when neither sensation nor emotion exerts any very appreciable influence, and when volition apparently exerts none at all. The movements in question seem to be quite as automatic—*reflex*, as it were—as those which spring from impressions made upon the spinal, sensory, or emotive ganglia. In the transition state between sleeping and waking, there is great fertility of incongruous thought—disorderly groups of ideas, receiving no governance whatever from the will; yet, in these circumstances, muscular movements and other phenomena will frequently take place, respondent purely to the dominant idea. An attractive object is before the imagination, and a snatch is made at it. Here there is no selection among motives—no will; the act is altogether impulsive, prompted by the simple idea. In certain irregular kinds of sleep, and in somnambulism spontaneously arising or induced by artificial processes, the mind can at times be literally *played upon*, so as to educe actions and movements contrived beforehand; these being suggested by communication of the correspondent idea, which becomes reflected in the outer conduct. Mr. Braid, in his hypnotic demonstrations, exhibits these phenomena in a very remarkable manner."

Dr. Noble adds (after detailing instances), that the phenomena constitute illustrations of Dr. Laycock's doctrine of the reflex functions of the brain. They have been termed by Dr. Carpenter (who, in the 4th edition of his very valuable "Elements of Human Physiology," has given great clearness and development to the automatic and unconscious activity of the cerebrum), *ideo-motor;* Dr. Noble prefers the term *ideo-dynamic*, as being more appropriate, and applicable to a wider range of phenomena. It may be doubted whether these phrases render the subject clearer. In his original Memoir,* Dr. Laycock endeavoured rather to demonstrate the relations to *quasi*-mental acts of changes in the brain itself, and not to states of consciousness particularly. The nature and order of occurrence of these changes in the organ of mind is the great unsolved problem of mental philosophy, and his object was to separate those which are not *necessarily* accompanied by consciousness, from those which are. He based his views on the anatomical and functional analogies that may be traced between the encephalic and spinal ganglia. Now, as in the posterior and anterior grey matter of the latter there is found a sensorial and a motor element, so he considered a sensorial and motor element (also constituted of vesicular neurine and conducting fibrils) to be present in the encephalic ganglia; and as in the spinal ganglia there is a play of vital affinities producing incident excitor and reflex motor phenomena, independently of consciousness, so in the encephalic there is a similar play of affinities. Dr. Noble quotes the following from Dr. Laycock's Memoir to illustrate his argument:—

"In this manner Dr. Laycock discusses the hydrophobic gasp, and after speaking of its induction by attempts to drink, traces the influence of mere idea in bringing about a like result. 'The cerebral nerves,' says he, 'being analogous to the posterior spinal nerves, and the encephalic ganglia analogous to the spinal ganglia, the spectrum of the cup of water will traverse the optic nerves, and enter the analogue of the posterior grey matter in the brain, causing changes (*ideagenous* changes) corresponding to the idea of water; thence the series of excited changes will pass over to the analogue of the anterior grey matter, exciting another series (*kinetic* changes) by which the necessary groups of muscles are combined in action.' The whole subject has also been

* On the Reflex Function of the Brain. British and Foreign Medical Review, Vol. XIX., January, 1845

admirably elucidated by Dr. Carpenter, in the last edition of his '*Human* Physiology.' "

Now, Dr. Noble has evidently not fully comprehended Dr. Laycock's views—misled, probably, by the phraseology necessarily used. We shall therefore help to a clearer apprehension by pointing out that in the above extract the *immediate* antecedent to the hydrophobic gasp is not the *idea* of water (for that implies consciousness), but the "*ideagenous*" and "kinetic" *changes* in the cerebrum—the changes by which ideas are *presented* to the consciousness, but not necessarily reaching it. It is these—the analogues of those incidial excitor changes in the spinal ganglia that induce acknowledged spinal reflex acts—which induce the cerebral reflex acts. The great object of Dr. Laycock's paper is to show that the cerebrum can and will act as automatically as the spinal cord, as independently of consciousness, as designedly, adaptively, and conservatively; and that even when consciousness is induced, whether in sensation or in thought, still it is not the will or sensation which is the principal agent of movement, but the *material changes* in the vesicular neurine or grey matter. The sensation or thought induced under these circumstances is only an *accompanying* and co-existing *result*, and not a necessary *antecedent*, or in the relation to the movements of a cause. He therefore argued that all these functions of the cerebrum could be, and often are, carried on unconsciously, and consequently occur in absolute independence of of the will. Whether they be right or wrong, these are his views; so that the phrase, reflex function of the brain (which has reference to cerebral anatomy and physiology), is convertible into that of unconscious action of the brain, in reference to mental philosophy.

Nor did Dr. Laycock confine his doctrines to the nervous system of the *vertebrata*, for he argued, in this same Memoir, "That the structure and functions of the nervous system, in *all* animals, are subject to the same laws [illegible] development and action;* and, in accordance with this view, devote[illegible] a paragraph to a consideration of the histological or molecular re[illegible]tions of the nervous system to the *instinctive*, *emotional*, and *volitiona*[illegible] actions.† He went even further, for in expressing his conviction that the laws of action of the agent and reagent in vital phenomena would be found as definite as those operating on chemical phenomena, could a sufficiently minute analysis and induction be effected, he extended his views to the phenomena of *vegetable* instincts. In a previous publication, Dr. Laycock had drawn the inference that "no bio-molecular movements take place, even in animals of the highest organization, which have not their counterpart in vegetables and in

* Op. cit. p. 298.

† Ibid. p. 308 seq. "III.—The Substrata of Physical Phenomena."

animals of the lowest forms;"* and after a rapid review of vital and *quasi*-mental phenomena, as presented in series in vegetable and the ascending scale of animal organisms, remarked, that "the mode of action of the brain itself, as the organ of mind, may in some degree be ascertained by a microscopic analysis of the series of phenomena reviewed."†

Subsequently when, in a correspondence with the late Professor Reid and Mr. Combe, Dr. Laycock had to explain and defend his views as to cerebral action, he again called attention to these leading points, and thus announced the grand principle by which cerebral physiology and pathology can alone be successfully elucidated:—"*The development, conservation, and reproduction of all organisms are regulated by an unerring law of design—a law as generally applicable to living matter as the law of gravity to universal matter.* This law must be our guide in ascertaining the relations of the reflex, instinctive, emotional, and voluntary movements."‡ Other passages of similar import might be added, but these will suffice to show the true nature of Dr. Laycock's views.

Dr. Noble gives some interesting illustrations of the influence which peculiar ideas and trains of thought exert under circumstances in which volitional agency is imperfect or altogether in abeyance, as in insanity. Those are especially curious in which the cerebral changes thus induced influence the *sympathetic* system, as showing a closer and deeper connexion between the two systems than has yet been generally allowed. Dr. Noble was consulted by a patient who suffered from sleepless nights, and in the exercise of his discretion he prescribed a *purgative*, consisting of eight grains of compound extract of colocynth and two of calomel, to be taken at bedtime. The patient so took the purge, believing it to be hypnotic; and as a result, instead of being purged, "slept beautifully!" Dr. Noble justly observes, that instances of imaginary remedies producing the expected effect are common enough, but here was the action of a powerful medicine altogether altered. According to Dr. Laycock's views, the following would be an analogous instance of action in the sympathetic in one of the *Articulata*. The *cunning* of the spider tribe in the pursuit of prey and escape from danger is well known to naturalists. "A garden-house had been plastered with very white lime, and having a large window, was unusually light. In a short time some spiders of a common kind took possession of the corners of the ceiling, there being no holes or cavities in which they could hide themselves. They soon became as *white as the wall*, but very certainly not from the lime adhering to them."§ It is one of homeopath

* Treatise on the Nervous Diseases of Women, p. 93. † Ibid. p. 36.
‡ The *Lancet*, Vol. II. 1845, p. 256. § The Naturalist, August, 1854, p. 167.

the tricks of fence of these animals to conceal or render themselves as invisible as possible; indeed, in the next page of the magazine from which we have taken this fact, another is stated, of the large diadem spider rendering himself invisible by giving to himself and his web a rapid vibratory motion. We have therefore an instance in which the colour of the skin was adaptively changed by the predominant idea of the animal's instinct.

Dr. Noble, in this Lecture, discusses the physiology of the emotions in a very able and interesting manner; his remarks on the nature of the will and of consciousness are also clear and good. Indeed, as to all the three Lectures, we only do a simple act of justice in expressing a very favourable opinion; and we have the greater pleasure in doing this because, on a former occasion, we thought it right to criticise a work by Dr. Noble somewhat sharply. We do not pretend to be infallible; we may have been too severe in our judgments, but this we can say, that whatever may be the merits or demerits of his "Elements of Psychological Medicine"—and of those the public will judge, however critics may criticise—these Lectures indicate a decided improvement—more reading, maturer thought, clearer conceptions.

We observe that Dr. Noble has carefully read the excellent work of Crichton on "Mental Derangement." He will doubtless regret to find on a careful perusal of Unzer's "Physiology," that some of Crichton's principal merits are due to unacknowledged plagiarisms from the latter admirable treatise.

---

